# Special Issue “Plastic and Reconstructive Surgery in Personalized Medicine”

**DOI:** 10.3390/jpm13030569

**Published:** 2023-03-22

**Authors:** Raymund E. Horch, Andreas Arkudas

**Affiliations:** Department of Plastic and Hand Surgery and Laboratory for Tissue Engineering and Regenerative Medicine, University Hospital Erlangen, Friedrich Alexander University Erlangen-Nuernberg FAU, Krankenhausstrasse 12, 91054 Erlangen, Germany

With an ever-growing knowledge in various disciplines of medicine and with rapidly evolving new techniques and operative methods in plastic surgery, it is obvious that it becomes more and more difficult to keep up with all the developments in this field at any time. Despite a plethora of scientific literature in various media, we felt that a Special Issue on the trending topics in plastic and reconstructive surgery could help to gain an overview of the recent advances in the field. We have therefore attempted to bundle the latest research and clinical data in a Special Issue on plastic and reconstructive surgery which, as such, offers a broad spectrum of different contemporary reconstructive techniques, including split skin transplantation and local as well as free flaps. Due to worldwide increasing expertise today, by means of microsurgery and perforator flaps, almost every reconstructive issue can be addressed somehow utilizing individually tailored techniques. Flaps can by their very nature comprise different tissues and can be custom designed using new imaging technologies [1,2,3] in order to increase the safety of the procedures and to retain the form and function of the reconstructed area in accordance with the donor site morbidity [4], as well as to reduce complications [5]. Automated devices—such as topical negative pressure application—to clean and precondition complex wounds and make them suitable for flap or skin graft coverage have found their way into daily clinical practice [2]. As with other developments of the specialty, the ingenuity of plastic surgeons leads to a continuous further evolution, and improvements of such technical tools are subject to sustained improvement and new indications. Additionally, given the modern armamentarium of surgical options today, approaches can be adjusted to increasingly personalized surgical treatment.

This issue of the Journal of Personalized Medicine covers several trending topics, such as individualized microsurgery, flap imaging, customized perforator flaps, monitoring flap perfusion, tailored tissue engineering for reconstruction, and biofabrication applications in personalized plastic and reconstructive surgery. This also includes research into the background of what plastic surgeons do and how the science behind our operative treatments may alter or influence future developments [6,7,8,9]. This Issue demonstrates how scientific research and clinical alertness and experience merge into new knowledge to further our capability to help aid patients with reconstructive problems.

In detail, Grüner et al. describe their experience with topical negative pressure therapy with instillation to cope with infected alloplastic implants in breast surgery and offer a new concept for this clinical issue [10]. Promny and coworkers discuss their findings concerning the safety of lipotransfer after breast-conserving therapy (BCT) and irradiation in breast cancer patients. They highlight that the safety of lipotransfer has still not been clarified yet due to contradictory data, and they present an innovative approach to provide more scientific data to clarify the issue [11]. Polykandriotis et al. present their research on the mechanical properties of suture materials and studies of how sutures break down under cycling loading [12]. Luze and coworkers assess the viability of skin flaps with thermal imaging as a potential personalized approach [13]. Frank et al. show the improved safety of DIEP flap transplantation with a detailed perforator anatomy study [14]. Hsu et al. studied the number of surgical interventions and specialists involved in the management of patients with neurofibromatosis type I in a 25-year analysis and discuss their approach to provide comprehensive individualized care to patients with NF [15]. Geierlehner and coworkers investigated the intraoperative blood flow of DIEP vs. ms-TRAM flaps in breast reconstruction combining transit-time flowmetry and microvascular indocyanine green angiography to learn more about the various flow patterns and to establish a threshold for optimal anastomotic conditions [16]. Cai et al. successfully managed to establish a special microsurgical transplantation technique of pedicled muscles in an isolation chamber as a novel approach to engineering muscle constructs via perfusion decellularization in an animal model [17]. Bigdeli and coworkers demonstrate the value of the free myocutaneous tensor fasciae latae flap for sternal defect reconstructions in a single-center experience with a considerable number of patients [18]. In another study, they compared the use of combined versus single perforator propeller flaps for the reconstruction of large soft tissue defects in a retrospective clinical study [19]. Dastagir and coauthors describe their clinical algorithm for non-invasive and surgical modalities for scar management [20]. Müller-Seubert et al. demonstrate intra- and early postoperative evaluations of malperfused areas in an irradiated random pattern skin flap model using indocyanine green angiography and near-infrared reflectance-based imaging and infrared thermography [21]. The value of indocyanine green to control leakage in isolated limb perfusion is described as a new and effective tool by Zucal and coworkers [22]. In addition, Thiem et al. studied the value of hyperspectral imaging for the clinical assessment of free flap monitoring compared to clinical monitoring in a prospective non-randomized clinical trial [23]. Diana Heimes and coworkers also investigated hyperspectral imaging if it is suitable to assess collateral circulation prior to radial forearm free flap harvesting and compare this tool to the conventional Allen test [24]. A retrospective cohort analysis by Wagner et al. describes the detection of post-traumatic lymphedema after open fractures of the lower extremity [25].

The prospects of hyperspectral imaging are also highlighted by Nischwitz et al. who prospectively compared thermal, hyperspectral, and laser Doppler imaging as non-invasive tools to detect the deep inferior epigastric artery (DIEP) perforators [26]. On the basis of their huge experience with hundreds of pelvic reconstructions, Horch et al. described a very innovative approach using the transpelvic vertical rectus abdominis myocutaneous (VRAM) with a new modification to allow an individualized procedure, including the urethral orifice into the skin paddle of VRAM flaps (Figure 1) to circumvent urinary diversion and maintain an acceptable quality of life [27]. The use of a novel two-stage reconstruction technique for extended femoral bone defects using an allograft in accordance with the Capanna technique with an embedded vascularized fibula graft in an induced membrane according to the Masquelet technique is described by Combal and co-authors [28]. Another mode of free flap assessment is reported by Huang et al. who used a wireless bioelectrical impedance assessment system for the quantitative analysis of tissue status and potential vascular compromise following microsurgery [29]. Ehrl and co-authors address another clinically relevant problem—the challenging defect coverage after forequarter amputations—in a thorough review assessing different surgical approaches, including different flaps [30]. Adding another facete, Heinzel et al. review the interplay of psychosocial factors and peripheral nerve lesions with their carefully considered and interesting title “beyond the knife“ [31].

In summary, this Special Issue shows in an impressive broad spectrum of high-level clinical and basic scientific research which is currently ongoing in the field. The present compilation of multiple surgical techniques, innovative imaging, and research tools are highly recommended for anybody interested in advances and in an update on contemporary plastic and reconstructive surgery. It also highlights in an impressive way that plastic surgery is definitely a trending topic, from more general to highly personalized approaches. 

## Figures and Tables

**Figure 1:**
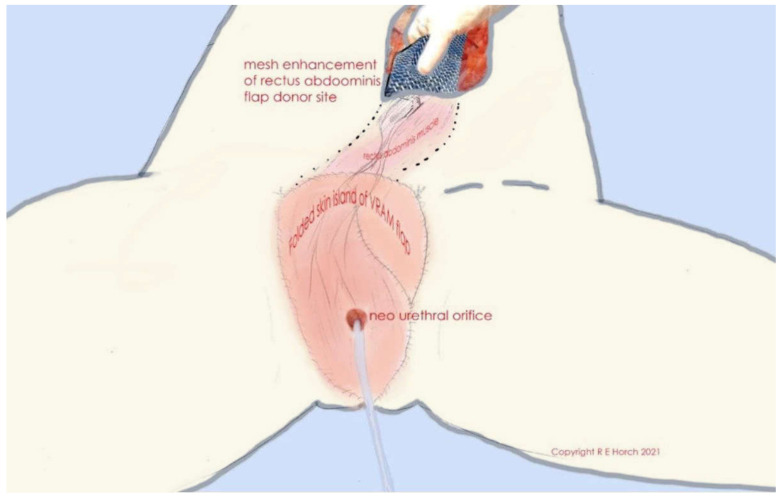
Schematic illustration of folded skin island of VRAM flap and neourethral orifice as well as of rectus abdominis muscle, tunneled subcutaneously. The flap donor site is closed with alloplastic mesh.
